# Synchronous double primary hepatocellular carcinoma and intrahepatic cholangiocarcinoma: A case report and review of the literature

**DOI:** 10.1097/MD.0000000000027349

**Published:** 2021-11-19

**Authors:** Meng-Meng Qu, Yuan-Hui Zhu, Yi-Xiang Li, Zhi-Fan Li, Jin-Kui Li, Yong-Sheng Xu, Manishkumar Shrestha, Jun-Qiang Lei

**Affiliations:** a The First Clinical Medical College of Lanzhou University, Lanzhou, China; b Department of Radiology, The First Hospital of Lanzhou University, Lanzhou, China.

**Keywords:** chronic liver infection, double primary hepatic cancer, hepatocellular carcinoma, imaging, intrahepatic cholangiocarcinoma

## Abstract

**Rationale:**

Presence of synchronous double hepatocelluar carcinoma (HCC) and intrahepatic cholangiocarcinoma (ICC) (sdpHCC-ICC) located separately within a single liver is extremely rare. The purpose of this study is to investigate the clinical, imaging, pathological characteristics, and prognosis of patients with sdpHCC-ICC, in order to enhance our understanding of the disease and improve diagnostic and therapeutic effect.

**Patient concerns:**

A 49-year-old, female with the diagnosis of hepatitis B virus with obvious liver cirrhosis, was admitted to our hospital. On admission, the levels of α-fetoprotein and carbohydrate antigen 19-9 were found to be elevated. Abdominal ultrasonography and enhanced computed tomography revealed 2 solid masses located in segments (S) 4 and 6 of the liver, with malignant behaviors.

**Diagnoses:**

In the light of above investigations, preoperative diagnosis of multiple primary hepatocellular carcinomas was made.

**Intervention:**

Hepatic resection of both segments was done. The resected specimens revealed the presence of well-defined tumors in segments 4 and 6 measuring 5.0 cm and 2.5 cm respectively.

**Outcomes:**

Histopathological examination confirmed the tumor of the 4th segment to be moderately and poorly differentiated ICC, and the tumor of the 6th segment to be poorly differentiated HCC. Immunohistochemically, the ICC in S4 was positive for CK19 and negative for Heppar-1, whereas the HCC in S6 was positive for Heppar-1 and negative for CK19. Unfortunately, metastasis to multiple organs and lymph nodes were observed 3 months later. The patient died of liver failure 16 months after surgery.

**Lessons:**

The clinical characteristics of sdpHCC-ICC are usually atypical and nonspecific making its preoperative diagnosis quite difficult. Hepatitis B virus and hepatitis C virus infection were both the independent risk factor for the development of sdpHCC-ICC. In patients with chronic liver disease, careful observation with imaging is of utmost necessity. Tumor markers may also play a valuable role in the diagnosis. The definite diagnosis depends on pathological examination. Hepatic resection is considered the most effective mode of treatment. The prognosis of synchronous occurrence of double hepatic cancers is worse than either HCC or ICC, and the origin of the disease needs further study.

## 1 Introduction

Primary liver carcinoma can histologically be divided into 3 types according to World Health Organization^[1]^ viz hepatocelluar carcinoma (HCC), intrahepatic cholangiocarcinoma (ICC), and combined hepatocellular carcinoma and cholangiocarcinoma (cHCC-CCA). Although HCC and ICC are the 2 main forms of primary liver cancer, the incidence of synchronous double primary hepatic cancers comprising both of them is very low.^[2–4]^ The 2 types of tumors can be recognized concurrently in the same tumor or separately at different sites in the same liver. Allen^[5]^ classified the tumors of concurrent HCC and ICC into 3 categories, which included type A (separate double nodules of HCC and ICC), type B (contiguous masses of HCC and ICC) and type C (the mixed tumor intermingling with components of both HCC and ICC). The latter 2 tumors are a form of cHCC-CCA according to the present consensus^[6]^; however, the former is separately regarded as synchronous double primary hepatocellular carcinoma and intrahepatic cholangiocarcinoma (sdpHCC-ICC). Due to the rarity of sdpHCC-ICC, only a few studies had been performed.^[2–4,7–9]^ Furthermore, the clinical understanding of the disease is poor, making the preoperative diagnose a challenge. In this case report, sdpHCC-ICC with a background of hepatitis B virus (HBV) infection and obvious liver cirrhosis will be discussed after the detailed review and analysis of the currently available literature of related clinicopathological and imaging features with the clinical diagnosis and treatment characteristics in order to improve the clinical understanding of sdpHCC-ICC.

## 2 Case presentation

A 49-year-old female was admitted in our hospital with the chief complaints of pain abdomen and weakness. Four years prior to the admission, she was diagnosed with hepatitis B infection with obvious liver cirrhosis. She was managed with antiviral therapy (entecavir) and anti-fibrosis therapy (soft-liver tablet of Trionyx nail) along with yearly physical examination and routine abdominal scan. She also had a history of uterine leiomyomas for several year and was treated with progesterone preparation.

**Figure 1:**
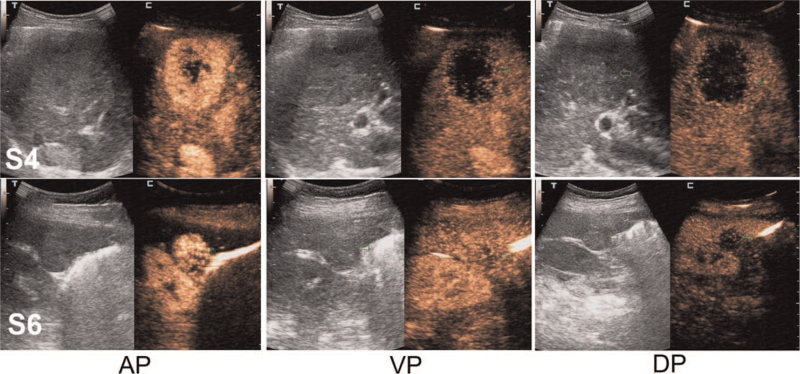
Abdominal contrast-enhanced ultrasonography (CEUS) identified 2 separate well-defined hypoechoic tumors that were heterogeneous on the inside in the segment 4 (S4) and segment 6 (S6) of the liver (green arrows; 5.0 × 4.6 cm and 2.5 × 2.1 cm in size, respectively). The S4 tumor presented high heterogeneous enhancement and the S6 tumor showed homogeneous enhancement in the arterial phase, The both tumors showed low enhancement in the portal venous phase and delayed phase. AP = arterial phase, DP = delayed phase, VP = portal venous phase.

**Figure 2:**
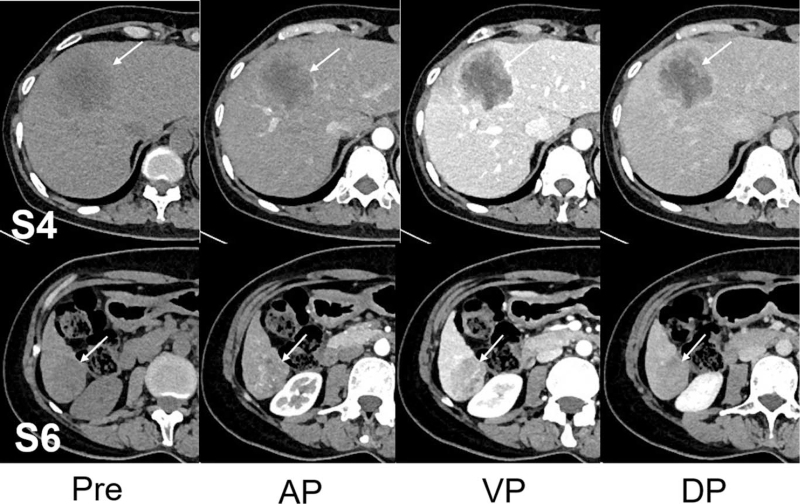
Abdominal computed tomography (CT) imaging of the 2 tumors (white arrows). Pre-contrast phase revealed 2 separate hypoattenuating hepatic masses in S4 and S6 (white arrows; 5.0 × 4.6 cm and 2.5 × 2.1 cm in size, respectively). On contrast-enhanced dynamic CT, the S4 tumor showed peripheral enhancement with central low density in the arterial phase and the peak enhancement appeared in the portal venous phase, and presented slightly centripetal enhancement in the delayed phase. The whole S6 tumor was heterogeneously enhanced in the arterial phase and followed by wash-out in the portal venous phase, and the delayed phase. AP = arterial phase, DP =  delayed phase, Pre = pre-contrast, VP = portal venous phase.

**Figure 3:**
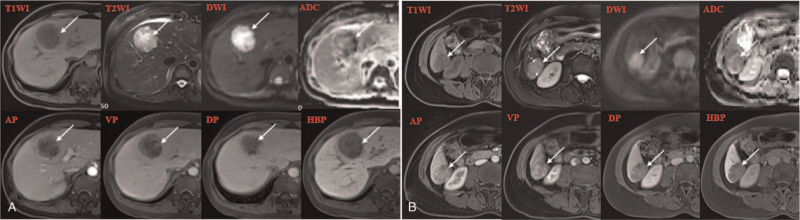
Abdominal magnetic resonance findings of the 2 tumors (white arrows). The tumors in S4 (A) and S6 (B) indicated similar findings of low signal intensity on T1WI and high signal intensity on T2WI. The DWI (b = 800 mm^2^/s) showed high-intensity masses and ADC map from conventional DWI showed the massed with decreased signal intensity, suggesting both tumors diffusion restriction. The S4 tumor showed rim-enhancement throughout arterial, portal venous, and delayed phase. The S6 tumor showed arterial-enhancing and delayed wash-out. The hepatobiliary phase appeared as hypointense nodules. ADC = apparent diffusion coefficient, DWI = diffusion weighted imagings, HBP = hepatobiliary phase, T1WI = T1-weighted images, T2WI = T2-weighted images.

She was subjected to abdominal contrast-enhanced ultrasonography which revealed the presence of 2 separate well-defined hypoechoic masses in the segment 4 (S4) and segment 6 (S6) of the liver which measured 5.0 × 4.6 cm and 2.5 × 2.1 cm in size, respectively. The tumors were heterogeneous on the inside and had enhanced characteristics, suggesting that both of them were hyper vascular in nature (Fig. 1). Subsequently, preoperative abdominal contrast-enhanced computed tomography (CT) and magnetic resonance imaging (MRI) were performed which provided the images of 2 separate masses with different contrast features (Figs. 2 and 3). Based on these preoperative examinations, the liver tumors were diagnosed as atypical multiple primary hepatocellular carcinomas. Further laboratory investigation included: platelet and white blood cell counts of 76.00 × 10^9^/L and 2.91 × 10^9^/L, respectively; hemoglobin, albumin, and total bilirubin levels of 120 g/L, 52.4 g/L and 17.3 μmol/L, respectively; and aspartate (AST) and alanine aminotransferase (ALT), alkaline phosphatase, and gamma-glutamyl transpeptidase concentrations of 34.6 U/L, 26.0 U/L, 129.4 U/L, and 48.4 U/L, respectively. She had a prothrombin time (percent) of 57.0%. Her indocyanine green retention rate at 15 minutes was 1.3%. Hepatitis B virus antigen was positive. Her serum alpha-fetoprotein was elevated (82.7 IU/mL, normal <5.8 IU/mL). The level of carbohydrate antigen 19–9 was also found to be elevated (270.6 U/mL, normal <27 U/mL).

The patient underwent partial liver resection with cholecystectomy. The resected specimen revealed the tumor in S4 as a well-defined off-white slightly hard elastic lesion, and the tumor in S6 as a round yellowish-white soft elastic lesion. Intraoperative pathologic examination of a lymph node at the hepatic portal region was performed concurrently which identified the presence of tumor cells in the lymph node which warranted the addition of extensive lymphadenectomy to the previous procedure.

**Figure 4:**
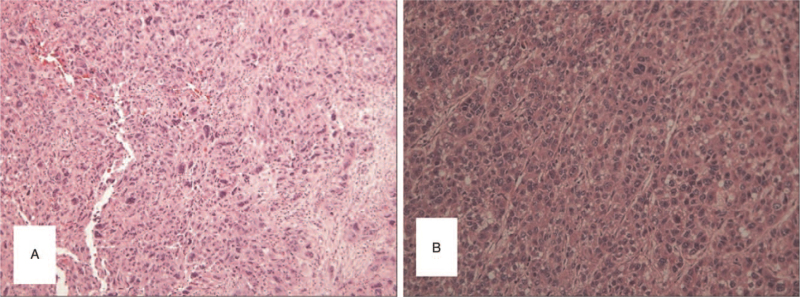
The histopathological findings of the 2 liver tumors (hematoxylin and eosin staining, ×100). The S4 tumor was moderately and poorly differentiated intrahepatic cholangiocarcinoma (A). The S6 was poorly differentiated hepatocellular carcinoma (B).

The pathological examination revealed the tumor in the S4 as moderately and poorly differentiated ICC (Fig. 4A), while that of the S6 as poorly differentiated HCC (Fig. 4B). No traces of cancerous emboli were found in vein and lymph vessel. The hepatic tissue adjacent to the tumors were found to be cirrhotic during pathological examination. During the immunohistochemical examination, the S4 tumor cells stained positive for CK19 and negative for Heppar-1, whereas the S6 tumor cells stained positive for Heppar-1 and negative for CK19. According to the histological findings, the liver tumors in this patient were diagnosed as synchronous double primary liver cancers.

The postoperative course was uneventful and she was discharged on postoperative day 15. During the first follow-up visit that was scheduled a month after the discharge, there were evidences of new recurrent lesions in the left lobe of the liver. So transcatheter arterial chemoembolization (TACE) was performed after checking for the presence of any contraindications. Unfortunately, the test confirmed the presence of recurrence along with metastasis in bilateral lung, left pubic bone, lymph nodes in the hepatoduodenal ligament and the Para aortic region. The patient died of liver failure 16 months after surgery.

## 3 Discussion and conclusions

sdpHCC-ICC is a rare and special type of primary liver malignancy whose pathogenesis is still unclear. HCC and ICC both have potential to occur independently. The primary tumor to occur may be HCC which may transform to ICC or vice versa. The malignant transformation of hepatic progenitor cells that occur may differentiate completely or incompletely into HCC and ICC.^[10]^ In some previous reports, sdpHCC-ICC was generally included as cHCC-CCA.^[5,11,12]^ However, the World Health Organization (WHO) reclassified cHCC-CCA in 2004, and called independently occurring HCC and ICC simultaneously in the liver as double primary carcinoma, which were excluded from cHCC-CCA. The proportion of sdpHCC-ICC is usually much lower than that of cHCC-CCA. A previous retrospective study reported that sdpHCC-ICC occupied 0.23% of primary liver cancers^[8]^ while the Surveillance, Epidemiology, and End Results (SEER) database showed the actual incidence of sdpHCC-ICC to be far less than 0.8%.^[13]^ Due to its rarity, it is very difficult to diagnose the disease before the pathological confirmation of the surgical specimens. None of the 10 cases were diagnosed accurately preoperatively in the above-mentioned retrospective study.^[8]^ In fact, Inaba et al^[2]^ indicated that most cases of multiple liver tumors were diagnosed initially as HCCs, with only about 20% being accurately diagnosed as synchronous primary hepatic tumors. The case of our report was also misdiagnosed as multiple HCCs prior to pathological confirmation.

There have been reports that noted a relationship between chronic liver inflammation and sdpHCC-ICC, as most cases suffered from chronic hepatitis or cirrhosis.^[2,4]^ It is a well-recognized fact that HBV or hepatitis C virus (HCV) infection leads to hepatitis and liver cirrhosis which are potential risk factors for HCC and ICC.^[14–16]^ Moreover, these relationships were supported by previous studies which showed that chronic liver inflammation played a significant role at the molecular level in coincidental double primary tumor that are HCC and ICC.^[17,18]^ Among these, HCV infection may be considered to have one of the closest associations with development of sdpHCC-ICC, as most of cases with the disease of interest had had a prior HCV infection.^[2,19,20]^ For example, the report by Watanabe et al^[19]^ which included 33 synchronous double cancer cases confirmed that 72.7% of patients had HCV infection, whereas only 9.3% of patients had HBV infection. However, the subject of our study, the 49-year-old female had a history of HBV infection with obvious cirrhosis of the liver.

The clinical manifestations of HCC and ICC are not specific, and only a few patients present with symptoms like discomfort and fatigue in earlier course of the disease, which makes the early accurate clinical diagnosis difficult. In practice, blood tumor markers and imaging findings are important tools to determine the diagnosis of primary liver cancers. Heppar-1 and α-fetoprotein (AFP) are considered as the most significant tumor marker for HCC, whereas CK7 and carbohydrate antigen 19-9 (CA19-9) are valuable markers for differentiating ICC from HCC.^[21,22]^ According the study of Cao,^[23]^ the frequency of simultaneous increment of AFP and CA19-9 levels in sdpHCC-ICC (29%) was significantly higher than that in pure HCC (9%) or ICC(6%). This may be an important characteristic of sdpHCC-ICC, and might help to preoperatively distinguish multiple HCCs or ICCs from sdpHCC-ICC. In our report, the simultaneously elevation for both AFP and CA19-9 levels may provide some information. However, the tumor markers are neither sensitive nor specific enough to diagnose sdpHCC-ICC. As cHCC-CCA has the components of both HCC and ICCs, the elevation of 2 tumor markers can also be observed, due to which the need of imaging examination is of utmost important to help in further diagnosis. Recently, some studies have examined the imaging findings in cases of ICC.^[24–26]^ Ultrasound usually shows hypoechoic mass. However, characterization of a malignant mass on US is often limited because of its variability and nonspecificity.^[27]^ CT and MRI with the aid of various types of contrast enhancement reveal the tumor as a well-defined solid tumor.^[24,26]^ The main feature of ICC is “delayed reinforcement”, which appear as a peripherally enhanced lesion during the early phase followed by a mild centripetal progression of enhancement over time in dynamic CT and MRI.^[28]^ The preoperative CT or MRI findings always diagnose the tumor as “atypical liver cancer” and is unable to give an accurate diagnosis in many cases.^[19]^ The primary mass feature observed in enhanced CT or MRI image is “fast wash-in and fast wash-out”, which is the characteristic feature of HCC. But the secondary masses were always considered to be the satellite nodules or intrahepatic metastasis of primary tumor. So, clinicians can make a diagnosis of multiple liver malignant tumors for sdpHCC-ICC patients. In fact, the CT or MRI images of sdpHCC-ICC are mainly the combinations of the images that are characteristics of both HCC and ICC due to which clinicians should consider the possibility of sdpHCC-ICC even if its incidence is very low when such imaging phenomenon is witnessed. The review of the CT and MRI features of our patient, showed that, although the larger tumor had several features of ICC, it was still difficult to confirm the diagnosis. In this case, the diagnosis might have been further confused by the knowledge of a higher prevalence of HCC than ICC in HBV patients. Therefore, for patients with simultaneous increase of AFP and CA19-9, sdpHCC-ICC should be strongly suspected especially in patient with chronic hepatitis, even if the prevalence of it is very low. A variety of imaging methods should be combined to further improve the preoperative diagnosis of sdpHCC-ICC.

Several modes of treatment for the patients with sdpHCC-ICC are available. Among these, surgical resection is regarded as the treatment of choice because it can be both diagnostic and curative especially in the patient without cirrhosis.^[29,30]^ The selection of candidate ideal for surgery is of utmost importance. The important factors that play the vital role for selection are pre-existing cirrhosis and tumor extent^[31,32]^ since the tumor associated with liver cirrhosis has increased risk of serious postoperative complications, such as seroperitoneum, hypoproteinemia, and pleural effusion. These complications can be prevented with active protein supplementation and diuresis after the operation.^[33]^ Recently, aggressive treatments including liver transplantation have been used on patients as a radical choice^[34]^; but the long-term curative effect of liver transplantation need to be studied further. Transcatheter arterial chemoembolization, percutaneous ethanol injection, and radiofrequency ablation, after consideration of size and location of the tumors, can be used for unresectable and recurrent masses. However, sdpHCC-ICC contains more fibrous tissue and fewer vascular components than HCC, and therefore, the therapeutic effect of TACE or percutaneous ethanol injection is limited.^[35]^ Our case was subjected to hepatic resection for the primary tumor followed by TACE after an interval of 45 days for intrahepatic recurrence. The patient also showed evidence of metastasis and new recurrent lesions.

The prognosis of double primary cancer varies among different studies. However, the prognosis of sdpHCC-ICC has commonly been recognized to be poorer than that of either HCC or ICC due its ability to recur and metastasize readily.^[36]^ Cao retrospectively studied the clinical characteristics of 35 cases of sdpHCC-ICC and revealed that tumor recurrence developed in 77.1% of patients, and distant metastases were detected in 20% of patients after partial hepatectomy. The median overall survival (OS) was 18 months, and the 1, 3, 5-year OS rates were 60.0%, 28.9%, and 23.1%, respectively.^[23]^ Multivariate analysis showed that the tumor size, histological differentiation of the ICC component and presence of lymph mode metastasis were independent factors that affected OS. In addition, Li et al^[33]^ also noted in their study that the ICC tumor size can affect not only patient's disease-free survival, but also OS, whereas HCC tumor size can only effect OS. We speculated that the influence of ICC on disease-free survival, OS or even the overall situation of patients with sdpHCC-ICC was greater than that of HCC. Therefore, for patients with sdpHCC-ICC, the clinician may need to pay more attention in the development and management of the ICC.

In summary, we have reported an extremely rare case of simultaneous double primary liver cancer consisting of HCC and ICC at separate locations of the liver in a patient infected with HAB with chronic hepatitis. Although sdpHCC-ICC is very rare, the occurrence of it should be considered in the differential diagnosis of multiple liver tumors. The combination of tumor markers analysis and imaging findings may be helpful to determine an accurate preoperative diagnosis, but the final diagnosis depends on pathological and immunohistochemical examination. Surgery remains the primary treatment option. The prognosis of patients with sdpHCC-ICC is poor, and the ICC component plays a more significant role than the HCC component in influencing prognosis. Since chronic liver inflammation may give rise to double primary liver cancer, it is of much importance that more attention is to be paid in such patients during their management so that, sdpHCC-ICC, if developed can be caught early and accurately. More information about patients with such cases need to be collected for further research.

## Acknowledgments

We would like to thank all members of our group.

## Author contributions

**Conceptualization:** Jun-Qiang Lei, Meng-Meng Qu, Jin-Kui Li, Yong-Sheng Xu, Manishkumar Shrestha.

**Data curation:** Meng-Meng Qu, Yuan-Hui Zhu, Yi-Xiang Li, Zhi-Fan Li.

**Formal analysis:** Meng-Meng Qu, Yuan-Hui Zhu, Yi-Xiang Li, Jun-Qiang Lei, Manishkumar Shrestha.

**Funding acquisition:** Jun-Qiang Lei, Jin-Kui Li, Yong-Sheng Xu.

**Investigation:** Meng-Meng Qu, Jin-Kui Li, Yuan-Hui Zhu, Yi-Xiang Li, Zhi-Fan Li.

**Methodology:** Meng-Meng Qu, Jun-Qiang Lei, Yuan-Hui Zhu.

**Project administration:** Meng-Meng Qu, Jin-Kui Li.

**Supervision:** Jun-Qiang Lei, Yi-Xiang Li, Zhi-Fan Li, Yong-Sheng Xu, Jin-Kui Li, Manishkumar Shrestha.

**Validation:** Meng-Meng Qu, Jun-Qiang Lei, Yuan-Hui Zhu, Yi-Xiang Li, Jin-Kui Li, Zhi-Fan Li, Yong-Sheng Xu, Manishkumar Shrestha.

**Visualization:** Meng-Meng Qu, Yuan-Hui Zhu, Jin-Kui Li, Yi-Xiang Li, Zhi-Fan Li, Yong-Sheng Xu, Manishkumar Shrestha.

**Writing original draft:** Meng-Meng Qu, Yuan-Hui Zhu, Yi-Xiang Li, Jin-Kui Li, Zhi-Fan Li.

**Writing review & editing:** Jun-Qiang Lei, Jin-Kui Li, Yong-Sheng Xu, Manishkumar Shrestha.
